# Usefulness of plasma and apolipoprotein B-depleted serum samples in paraoxonase 1 assessment^☆^

**DOI:** 10.1016/j.bbrep.2025.102274

**Published:** 2025-09-21

**Authors:** Rina Kawaguchi, Akira Yoshimoto, Takahiro Kameda, Ryunosuke Ohkawa

**Affiliations:** a Clinical Bioanalysis and Molecular Biology, Graduate School of Medical and Dental Sciences, Institute of Science Tokyo, 1-5-45 Yushima, Bunkyo-ku, Tokyo, 113-8510, Japan; b Department of Clinical Laboratory Science, Faculty of Medical Technology, Teikyo University, 2-11-1 Kaga, Itabashi-ku, Tokyo, 173-8605, Japan

**Keywords:** Apolipoprotein B-depleted serum, Enzymatic activity, High-density lipoprotein, Paraoxonase 1, Sample preparation

## Abstract

Paraoxonase 1 (PON1) is closely associated with antioxidant, anti-inflammatory, and antiatherosclerotic functions of high-density lipoprotein (HDL). Although many clinical studies have evaluated relationships between PON1 activity and various diseases based on its multiple functions, their results were contradictory because of the difference of sample preparation methods. Therefore, we investigated an optimal preanalytical method for PON1 analysis by measuring three different PON1 activities in various types of specimens. Samples were prepared from healthy human serum, plasma with or without calcium addition, HDL isolated by ultracentrifugation, and apolipoprotein B-depleted serum (BDS). Using these samples, PON1 protein concentration and activities using three substrate types (*p*-nitrophenyl acetate, paraoxon, and γ-thiobutyrolactone) were evaluated. PON1 distributions in HDL subfractions from serum and BDS were also investigated. Although PON1 activities in plasma were lower than those in serum, removing EDTA and adding calcium rescued PON1 activities in plasma similar to levels comparable to those in serum. In contrast, HDL isolated by ultracentrifugation had significantly lower PON1 activities and protein concentrations, indicating that many PON1 proteins were not bound to the HDL particle in the HDL fractions collected from serum and plasma by ultracentrifugation. PON1 protein concentration and distributions in BDS showed similar to those in serum sample than those in HDL sample. Furthermore, three types of PON1 activities were differentially affected by sample preparation procedures. The reduction of PON1 activity in BDS differed among individuals and by the activity type. Focusing on each of three different PON1 activities might further enhance the clinical significance of PON1 testing.

## Highlights

• Plasma PON1 activity is maintained with immediate calcium supplementation.
• Ultracentrifugation-isolated HDL shows reduced PON1 protein concentrations.
• Apolipoprotein B-depleted serum preserves PON1 distribution similar to serum.
• Three PON1 activities are differentially affected by sample preparation procedures.

## 1 Introduction

Paraoxonase 1 (PON1), a high-density lipoprotein (HDL)-associated enzyme, is increasingly recognized for its crucial role in protecting against oxidative stress and inflammatory processes, particularly in the context of cardiovascular diseases. PON1, associated with HDL, hydrolyses lipid hydroperoxides (LOOHs) scavenged by HDL from oxidized low-density lipoprotein (LDL) [1,2]. Serum HDL-cholesterol (HDL-C) concentration has been measured as a biomarker for atherosclerosis in clinical test. However, HDL-C does not always reflect the HDL function because no interventional study targeting HDL-C elevation has shown the expected potency in the treatment of atherosclerosis [3]. Therefore, not only the quantitative assessment but also assessment of the HDL functions has gained renewed interest. In addition to the suppression of LOOH accumulation, PON1 contributes to various HDL functions, such as inhibiting the release of inflammatory factors from macrophages [4] and promoting a cholesterol efflux via an ATP-binding cassette transporter A1 [5]. Thus, PON1 is also considered as a clinical parameter reflecting the HDL multiple functions, and many studies have investigated associations of PON1 with a wide range of diseases, including atherosclerosis, cancer, and other infectious diseases [[6], [7], [8], [9]].

However, there are inconsistencies in sample preparation methods for assessing PON1. In lipoprotein studies, EDTA plasma is commonly used to avoid the copper-induced lipoprotein oxidation. Some studies have also used the EDTA plasma for studying PON1. PON1 is a calcium-dependent enzyme, and the use of EDTA plasma, which chelates calcium, can lead to suppressed or inconsistent enzyme activity measurements, thereby yielding unreliable results [10,11]. Regarding lithium heparin plasma, PON1 values were not comparable to those in serum as described previously [12].

In addition, PON1 hydrolyses various types of substrates such as aryl esters, organophosphorus, and lactones. Clinical studies that evaluate the usefulness of PON1 through the measurement of various PON1 activities, such as arylesterase, paraoxonase, and lactonase, respectively. Depending on the measured activity, different research results may be observed. Therefore, since the most suitable type of activity remains controversial [13,14], the PON1 activity should be evaluated using several types of substrates.

Consequently, the differences in the sample collection methods and substrate selection might be the reasons for the conflicting results of PON1 studies; some studies have reported inverse correlations between PON1 activity and cardiovascular disease outcomes [[15], [16], [17]], while other studies dispute the association [15,18,19].

Serum and HDL, which are currently used as specimens, also have problems in conducting PON1 studies. Notably, PON1 is known to detach from the HDL particle during ultracentrifugation, which means studies using HDL fractions may not accurately replicate the *in vivo* PON1-associated environment [20]. In addition, PON1 is transported to the other apolipoprotein B-containing lipoproteins such as LDL from HDL [21]. Although serum PON1 studies allow comprehensive PON1 investigations, these might be insufficient for targeting only HDL-PON1 (HDL functions). Apolipoprotein B-depleted serum (BDS), which can exclude the effects of apolipoprotein B-containing lipoproteins without ultracentrifugation, is used as a specimen for HDL functional study [22]. This method offers a significant advantage over ultracentrifugation, which is known to dissociate PON1 from HDL particles, thus failing to accurately represent the *in vivo* PON1-associated environment. Despite the recognized advantages of BDS in assessing HDL function, its potential utility as an optimal specimen for PON1 research has remained largely unexplored.

Therefore, this study aimed to systematically investigate the impact of different sample preparation procedures, including calcium-supplemented EDTA plasma and BDS, on various PON1 activities. In addition, our goal of this study was to establish more accurate pre-analytical methods for PON1 assessment.

## 2 Materials and methods

### 2.1 Blood samples

Blood samples were collected from three healthy volunteers after obtaining their signed informed consent at Tokyo Medical and Dental University. The present study was approved by the ethics committee of the Faculty of Medicine, Tokyo Medical and Dental University (M2015-546). This study has been performed in accordance with the rules of the World Medical Association and the principles of the Declaration of Helsinki.

For serum preparation, blood was collected into plain collection tubes and allowed to clot at room temperature for 10 min, followed by centrifugation at 2000×*g* for 10 min at 4 °C. For EDTA plasma preparation, blood was collected into EDTA-containing tubes and immediately centrifuged at 2000×*g* for 30 min at 4 °C. All other samples were aliquoted and stored at −80 °C until use. The collected blood samples (serum or EDTA plasma) were stored at 4 °C before using in comparative experiment between serum and plasma PON1 activities, and they were stored at −80 °C until use for the other experiments. Blood samples were used within a week.

### 2.2 Cholesterol and protein quantifications

Total cholesterol (TC) concentrations were measured using enzymatic test kit (Denka Co. Ltd., Tokyo, Japan). The protein concentrations were assayed using the Folin–Lowry method [23].

### 2.3 Calcium addition by dialysis

Calcium was added to the collected serum or plasma by dialysis against the PON1 buffer (2 mmol/L CaCl_2_, 50 mmol/L Tris-HCl, pH 7.4) at 4 °C. For plasma, centrifugation was performed at 2000×*g* for 10 min at 4 °C to remove the fibrinogens precipitated by removing EDTA and adding calcium.

### 2.4 Preparation of HDLs

HDL (d = 1.063–1.210 g/mL) was prepared from three types of specimens. Calcium was added to plasma by dialysis as stated above, and HDLs were isolated from the pooled serums or calcium added plasmas by ultracentrifugation at 417,000×*g* for 22 h at 4 °C as described by Havel et al. [24] with some modifications; specifically, to adjust the density, we used potassium bromide solution at the specific gravity without EDTA-2K, to prevent the re-chelation of calcium from the previously calcium-treated plasma, which would otherwise inactivate PON1. To investigate whether the detachment of PON1 protein from HDL during ultracentrifugation differs between serum and plasma samples, and to ascertain if EDTA-2K or the coagulation process causes such differences, we prepared a third type of HDL: ‘serum plus EDTA’. For the preparation of HDL from ‘serum plus EDTA’, serum was initially subjected to ultracentrifugation to separate the very-low density lipoprotein (VLDL) and LDL fractions (d < 1.063 g/mL) from the lower fraction containing HDL and other plasma proteins. Immediately after this first ultracentrifugation step, EDTA-2K was added to the collected lower fraction (d > 1.063 g/mL) at a final concentration of 2.5 mM. This EDTA-2K-containing lower fraction was then subjected to a second ultracentrifugation to isolate the HDL fraction (d = 1.063–1.210 g/mL).

Finally, each of the HDL fraction isolated from serum, plasma, and serum plus EDTA was dialyzed against the PON1 buffer. The isolated HDL samples were stored at −80 °C. HDL samples were used within a month.

### 2.5 Preparation of BDS

BDS was prepared as described previously [25]. Briefly, 100 μL of serum was mixed with 40 μL of 20 % polyethylene glycol (PEG; 6000 Da) in 200 mmol/L glycine buffer (pH 7.4), followed by mixing and incubation at room temperature for 20 min. The mixture was centrifuged at 9170×*g* for 30 min at 4 °C, and the supernatant was isolated as BDS. HDL-C concentrations in BDS and the original serum were determined by measuring TC concentrations in BDS sample, and HDL protein concentrations in BDS and the serum were determined using protein–cholesterol ratio of the same individual's HDL isolated by ultracentrifugation.

### 2.6 PON1 arylesterase activity

Arylesterase activity was determined following a previously described method [26] with some modifications. Briefly, 13 μL of sample was mixed with 57 μL of arylesterase buffer (1.3 mmol/L CaCl_2_, 100 mmol/L Tris-HCl, pH 8.0) and 30 μL of 2 mmol/L *p*-nitrophenyl acetate (Fujifilm Wako Pure Chemicals Corporation, Osaka, Japan) dissolved in 90 mmol/L Tris-HCl (pH 8.0). Initial velocities of hydrolysis were determined at 405 nm using a SUNRISE Rainbow microplate reader (Fujifilm Wako Pure Chemicals Corporation, Osaka, Japan). The E_405_ for the reaction was 18,050 mL/mmol/cm.

### 2.7 PON1 paraoxonase activity

Paraoxonase activity was determined by following a previously described method [27]. Briefly, 40 μL of sample was mixed with 30 μL of paraoxonase buffer (2.63 mol/L NaCl, 1.3 mmol/L CaCl_2_, 50 mmol/L Tris-HCl, pH 8.5) and 30 μL of 2 mmol/L paraoxon (Toronto Research Chemicals Inc., Toronto, Canada) dissolved in 50 mmol/L Tris-HCl (pH 8.5). Initial velocities of hydrolysis were determined at 405 nm. The E_405_ for the reaction was 18,050 mL/mmol/cm.

### 2.8 PON1 homocysteine-thiolactonase activity

Homocysteine-thiolactonase activity was determined following a previously described method [28]. Briefly, 20 μL sample was mixed with 40 μL of 1 mmol/L 5,5′-Dithiobis-(2-nitrobenzoic Acid) (Fujifilm Wako Pure Chemicals Corporation, Osaka, Japan) and 40 μL of 50 mmol/L γ-thiobutyrolactone (Sigma-Aldrich Japan LLC, Tokyo, Japan) in the PON1 buffer (pH 7.4). Initial velocities of hydrolysis were determined at 405 nm. The E_405_ for the reaction was 14,150 mL/mmol/cm.

### 2.9 Western blotting

Western blotting (WB) was conducted following a previously described method [29]. HDL (adjusted to 2 μg HDL protein/lane), serum and BDS samples (adjusted to 0.4 μg of their respective HDL protein content/lane) were loaded on 10 % sodium dodecyl sulphate polyacrylamide gel electrophoresis (SDS-PAGE). Similarly, the samples (5 μg HDL protein/lane) were analysed by the electrophoresis using 7 % non-denaturing polyacrylamide gel electrophoresis (Native-PAGE). DynaMarker® Protein MultiColor (BioDynamics Laboratory Inc., Tokyo, Japan) was used as a standard molecular weight marker for SDS-PAGE. Calibration Kit For Native Electrophoresis (Cytiva, Tokyo, Japan) was used as a standard particle size marker for Native-PAGE [[30], [31], [32]]. Immunoblotting was conducted using the following antibodies: polyclonal rabbit *anti*-PON1 antibody (Sigma-Aldrich Japan LLC, Tokyo, Japan), monoclonal rabbit *anti*-PON1 antibody (Abcam plc., Cambridge, UK), and polyclonal goat anti-apolipoprotein A-I (apoA-I) antibody (Academy Bio-Medical Company Inc., Houston, TX, USA) as primary antibodies and polyclonal goat anti-rabbit IgG (Horseradish peroxidase [HRP]) (Abcam plc., Cambridge, UK) and polyclonal rabbit anti-goat IgG (HRP) (Medical & Biological Laboratories Co. Ltd., Tokyo, Japan) as secondary antibodies. The bands were visualized using ECL Prime Western Blotting Detection Reagents (GE Healthcare, Tokyo, Japan) or, for specific experiments, by incubating the membranes with 3,3′-diaminobenzidine tetrahydrochloride and hydrogen peroxide solutions. Semiquantification of each band was performed by densitometry on a CS Analyzer 4 (ATTO, Tokyo, Japan).

### 2.10 Statistical analyses

We used one-way analysis of variance with Bonferroni correction or Games-Howell correction to compare the results using SPSS ver. 25.0 (Chicago, Armonk, NY, U.S.A.). The results are expressed as means ± SD. A *P* < 0.05 was considered statistically significant.

## 3 Results

### 3.1 Prevention of PON1 deactivation in plasma by calcium treatment

Although EDTA is commonly used in plasma collection to prevent coagulation and inhibit copper oxidation [33], its strong calcium-chelating properties pose a challenge for the analysis of PON1, a calcium-dependent enzyme. Therefore, plasma samples are not directly suitable for accurate PON1 activity analysis without further treatment. We performed dialysis on plasma samples to both remove EDTA and simultaneously add calcium (calcium treatment), and subsequently investigated its effect on PON1 activities. To assess the long-term impact of calcium treatment on PON1 activity and considering previous findings on PON1 stability in EDTA [34], samples were analysed after storage at 4 °C for one week following dialysis. Fig. 1 showed PON1 activities in serum and plasma samples (with or without calcium treatment), normalized to TC concentrations of 150 mg/dL to account for dilution effects from dialysis. In plasma samples without calcium treatment, PON1 activities were significantly reduced compared to those in serum. Specifically, arylesterase activity decreased by 64 %, paraoxonase activity by 100 % (indicating almost complete loss of activity), and homocysteine-thiolactonase activity by 41 % (Fig. 1A–C). In contrast, calcium treatment via dialysis effectively restored PON1 activities in plasma, resulting in only minor decrements compared to serum levels: arylesterase activity by 9 %, paraoxonase activity by 8 %, and homocysteine-thiolactonase activity by 6 %. These findings suggest that calcium treatment can largely overcome the suppressive effect of EDTA on PON1 activities in plasma. Furthermore, our observations revealed differential sensitivity of the three PON1 activities to calcium deficiency. The reduction in paraoxonase activity in untreated plasma was notably more pronounced (approximately 2- to 2.5-fold greater reduction) than that observed for arylesterase activity and homocysteine-thiolactonase activities. These results indicated a higher dependency of paraoxonase activity on calcium availability.

**Fig. 1:**
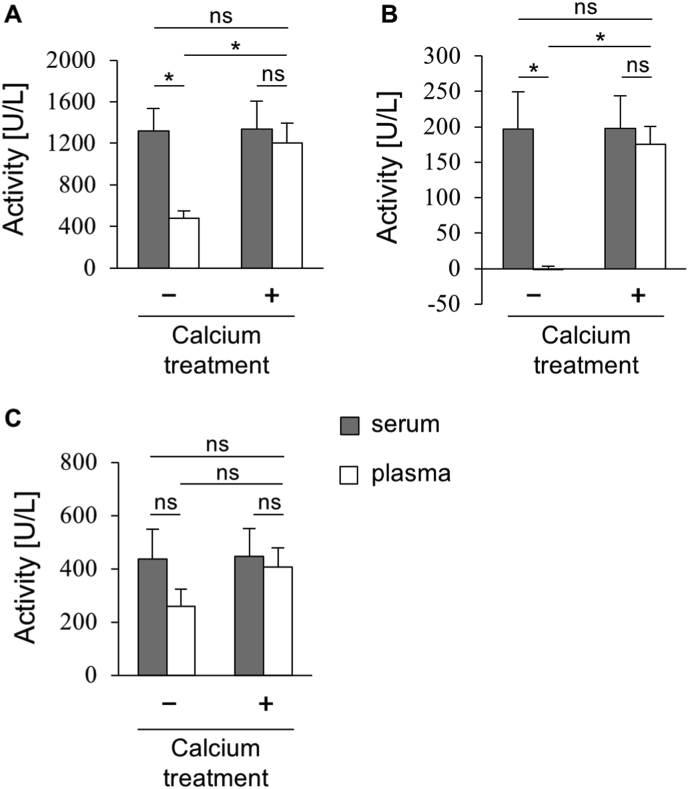
Effect of calcium treatment on serum and EDTA plasma PON1 activities. Serum and plasma samples were treated with or without calcium supplementation by dialysis against the PON1 buffer (2 mmol/L CaCl_2_, 50 mmol/L Tris-HCl, pH 7.4). After storing at 4 °C for one week, samples were assessed for three different PON1 activities: (A) arylesterase activity; (B) paraoxonase activity; (C) homocysteine-thiolactonase activity. PON1 activities were corrected to 150 mg/dL TC concentrations to account dilution due to dialysis. All samples were assayed in triplicate. Values are presented as means ± SD (n = 3). ∗*P* < 0.05 compared with the serum sample by one-way analysis of variance with Bonferroni correction (ns, not significant).

### 3.2 Comparison of PON1 activities between HDL samples isolated from serum, plasma, and serum plus EDTA

HDL is a critical source of PON1 enzymes in blood and is frequently utilized in PON1 studies. To investigate the applicability of plasma in HDL-PON1 studies and to delineate the distinct effects of EDTA and the coagulation process on PON1's association with HDL during ultracentrifugation, we prepared HDL samples isolated from plasma, serum, and serum with post-collection EDTA addition (termed ‘serum plus EDTA’). Fig. 2A–C shows the three different types of PON1 activities in the HDL fractions isolated from plasma, serum, and serum plus EDTA, normalized to the plasma sample as 1.0. While no statistically significant differences were observed among these groups, PON1 activities in HDL isolated from serum (e.g., Arylesterase activity: 2.2 ± 1.3 relative to plasma, mean ± SD; Fig. 2A) showed a tendency to be higher than those in HDL from plasma.

**Fig. 2:**
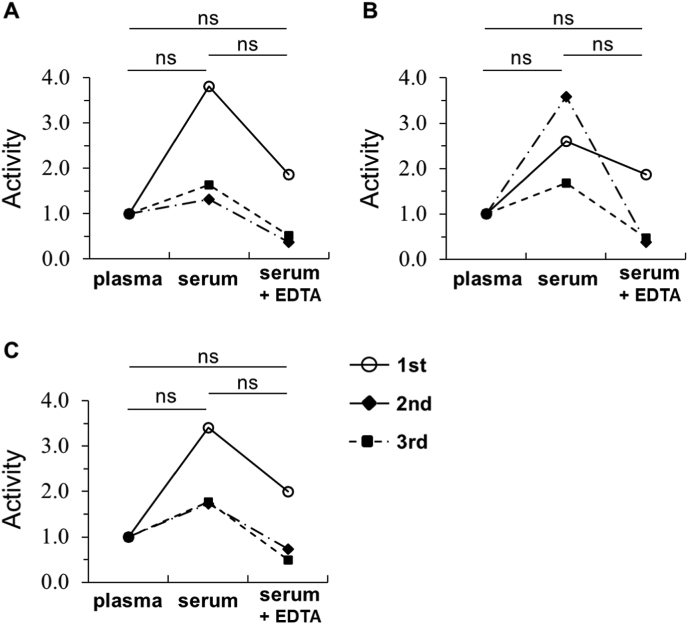
PON1 activities in the HDL fractions from plasma, serum, and serum plus EDTA. HDL was isolated from plasma, serum, and serum plus EDTA with the following treatment: Calcium was added to the collected plasma by dialysis before ultracentrifugation. EDTA was added to the part of serum sample between the first and second ultracentrifugation step. PON1 in each HDL (1.0 μg protein/mL) was assessed for three different types of PON1 activities: (A) arylesterase activity; (B) paraoxonase activity; (C) homocysteine-thiolactonase activity. All samples were assayed in triplicate from separate experiments. Relative values with an activity of HDL collected from plasma as 1.0 were shown (ns, not significant).

In contrast, significant differences were observed in PON1 protein concentrations (Fig. 3A). Specifically, HDL isolated from serum exhibited significantly higher PON1 protein concentrations compared to HDL from plasma. HDL from serum plus EDTA showed a similar level of PON1 protein concentration to that from plasma (Fig. 3A). The ratios of HDL-PON1 concentrations to that from plasma were 3.0 ± 0.39 for serum and 1.0 ± 0.78 for serum plus EDTA (Fig. 3B). Analysis of PON1 distribution by Native-PAGE revealed that a substantial portion of PON1 proteins were not bound to the main HDL particle peak even in the HDL fraction collected from serum (Fig. 3C). This suggests that a significant amount of PON1 may dissociate from HDL during the centrifugation process. Importantly, no discernible difference in the overall HDL particle sizes was observed among the samples (Fig. 3D), indicating that particle size itself did not account for the observed differences in PON1 association.

**Fig. 3:**
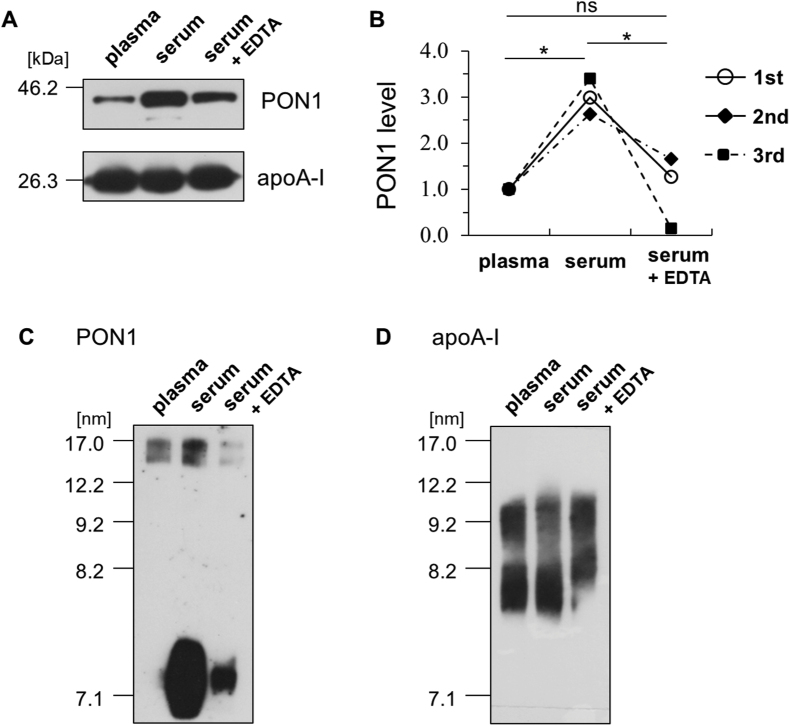
PON1 protein assessment in the HDL fractions isolated from plasma, serum, and serum plus EDTA. (A) SDS-PAGE and WB analysis of HDL samples (2.0 μg protein/lane) using *anti*-PON1 and -apoA-I. (B) Quantification analysis of PON1 per apoA-I bands. (C) (D) Resolution of HDL samples (5.0 μg protein/lane) by Native-PAGE and WB using *anti*-PON1 and -apoA-I antibodies. Each particle size number was indicated at the position where the following protein standards migrated: thyroglobulin (17.0 nm), ferritin (12.2 nm), catalase (9.2 nm), lactate dehydrogenase (8.2 nm), and albumin (7.1 nm). Representative profiles from three independent experiments are shown (A) (C) (D). ∗*P* < 0.05 compared with the HDL isolated from plasma sample by one-way analysis of variance with Bonferroni correction (ns, not significant).

### 3.3 Evaluation of PON1 activities and proteins in BDS

To assess the potential of BDS sample for HDL-PON1 study, we compared three types of PON1 activities and PON1 protein in serum, BDS, and HDL isolated from serum by ultracentrifugation obtained from three individuals. The PON1 activities in HDL isolated from serum by ultracentrifugation were significantly reduced compared to those in serum: arylesterase activity by −75 %, paraoxonase activity by −70 %, and homocysteine-thiolactonase activity by −83 % (Fig. 4A–C). Further, the PON1 protein concentration in HDL samples was notably lower, showing 99 % reduction compared to serum samples (Fig. 4D and E). Two distinct bands were observed for PON1 in HDL samples (Fig. 4D, lane 3). The upper band, which appeared to be around 46.2 kDa, is likely a contamination from the neighbouring lane (lane 2) due to the high concentration of PON1 in the adjacent samples. This was supported by the result that only a single band was detected below 46.2 kDa in the HDL fraction (Fig. 3A). On the other hand, multiple bands were detected in both serum and BDS samples (Fig. 4D, lanes 1 and 2). When this blot analysis was performed, the applied total protein amount of the serum sample was much higher than that in the HDL sample. However, both samples were treated with the same concentration of reducing agent (1.46 M 2-mercaptoethanol). Therefore, we re-confirmed the Western blot analysis of 16 serum samples under a higher reducing condition (2.5-fold 2-mercaptoethanol) and found that these multiple bands disappeared, suggesting their polymeric or aggregated nature (data not shown). These findings strongly indicate that the ultracentrifugation method causes a substantial release of PON1 from the HDL particle. In contrast, when comparing BDS samples to serum, each PON1 activity showed only slight, non-significant difference (Fig. 4A–C), with values normalized to serum as 1.0. However, it was observed that the reduction in PON1 activity in BDS varied among individuals and activity types. For example, the relative homocysteine-thiolactonase activities in BDS for three individual subjects were reduced by 0.17, 0.14, and 0.61, respectively, compared to serum (Fig. 4C). Furthermore, for subject C, the changes in the three activities in BDS relative to serum were distinct: arylesterase activity increased by 4 %, paraoxonase activity decreased by 14 %, and homocysteine-thiolactonase activity decreased by 61 % (Fig. 4A–C). These findings suggest that while BDS generally maintains PON1 activity comparable to serum, individual variations and activity-specific sensitivities exist.

**Fig. 4:**
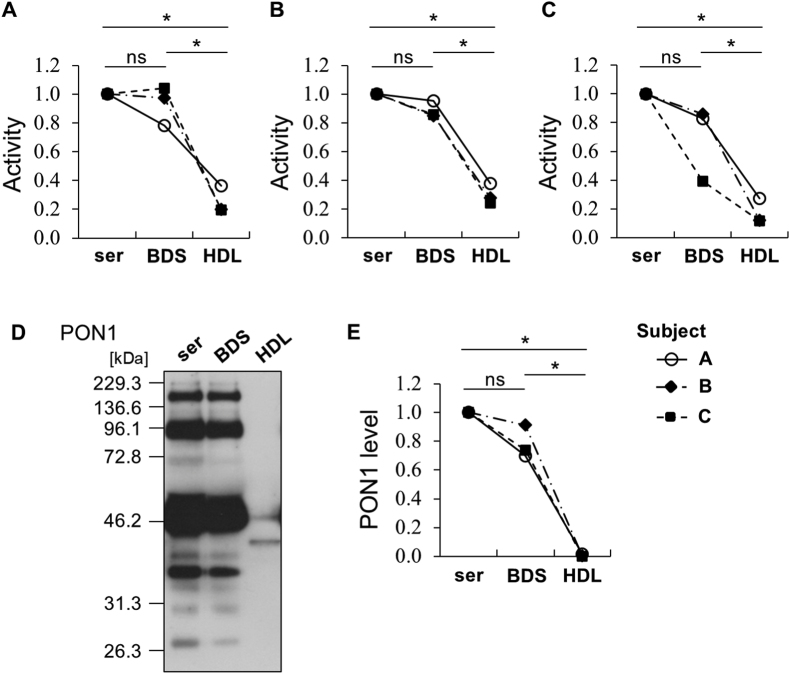
PON1 assessment in serum, BDS, and HDL samples collected from three individuals (Subject A, B, and C). PON1 in each HDL (1.0 μg protein/mL) was assessed for three types of PON1 activities: (A) arylesterase activity; (B) paraoxonase activity; (C) homocysteine-thiolactonase activity. Relative values with serum sample as 1.0 are shown. All samples were assayed in triplicate. (D) (E) HDL samples (2.0 μg HDL protein/lane), serum, and BDS samples (0.4 μg HDL protein/lane) were analysed by SDS-PAGE and WB using *anti*-PON1 followed by semiquantification analysis of PON1. Representative profile (Subject B) from three independent experiments is shown. ∗*P* < 0.05 compared with the serum sample by one-way analysis of variance with Games-Howell correction (ns, not significant; ser, serum; BDS, apolipoprotein B-depleted serum).

We observed that both PON1 activities and protein concentration in BDS samples remained largely similar to those in serum samples. The findings suggested that BDS can be effectively utilized for PON1 analysis, thereby avoiding the significant loss of PON1 proteins typically associated with ultracentrifugation. When preparing BDS, serum was mixed with PEG, followed by centrifugation. Although these manipulations are not generally expected to affect PON1 activities and concentrations, we considered the possibility that the binding of PON1 to the HDL particle might be altered. Therefore, we additionally investigated the PON1 distribution in BDS by Native-PAGE. PON1 protein distributions in BDS samples from three individuals were observed to be similar to those in serum samples (Fig. 5A). Comparing the profiles of PON1 with those of apoA-I, PON1 proteins were localized in some large HDL subclasses, consistent with observations in HDL collected by ultracentrifugation as shown in Fig. 3C. Notably, PON1 was also present on smaller HDL particles, specifically at sizes around 8.2 nm and approximately 7.3 nm, delineated as sharp and broad peaks, respectively, by densitometry (Fig. 5B). Crucially, unlike the HDL fraction obtained by ultracentrifugation, PON1 protein in serum and BDS, which were directly resolved by Native-PAGE, was not detected at the approximately 7.1 nm size position. This finding indicates that the manipulation involved in BDS sample preparation does not lead to the dissociation of PON1 from HDL particles, thus preserving the native association of PON1 with HDL.

**Fig. 5:**
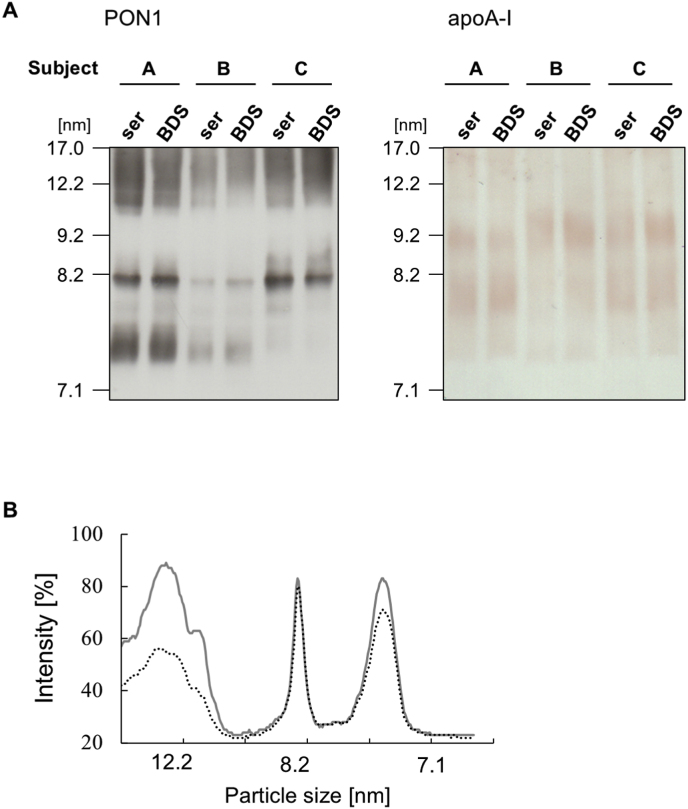
Comparison of PON1 distributions in between serum and BDS. (A) Serum and BDS samples from three individuals (Subject A, B, and C) were resolved by Native-PAGE and WB using *anti*-PON1 and -apoA-I antibodies. Each particle size number was indicated at the position where the following protein standards migrated: thyroglobulin (17.0 nm), ferritin (12.2 nm), catalase (9.2 nm), lactate dehydrogenase (8.2 nm), and albumin (7.1 nm). (B) Representative semiquantification profile of PON1 distribution in serum (solid line) and BDS (dashed line) delineated from Native-PAGE profile (Subject A) (ser, serum; BDS, apolipoprotein B-depleted serum).

## 4 Discussion

PON1 is one of the driving forces for HDL exerting various functions to regulate lipid metabolism [35]. Various cross-sectional studies have described the relationship between PON1 activities and various diseases [8,36]. However, there are few basic studies considering an approach for assessing the PON1 activity. We aimed to investigate a more suitable sampling procedure for studying PON1.

First, we investigated the use of EDTA plasma to PON1 assessment. EDTA, which is used for plasma preparation, chelates divalent cation including calcium to inhibit the clotting resulting in suppression of copper oxidation and the release of blood cell components. EDTA plasma has been widely used in lipoprotein studies, and thus we tried to use plasma sample for studying PON1. When EDTA plasma sample was used without any treatment, PON1 was deactivated because of irreversible denaturation of PON1 caused by calcium removal as shown in previous study [37]. Paraoxonase activity in plasma was completely inactivated, whereas homocysteine-thiolactonase activity was approximately half that in serum, indicating that deactivation varied depending on the type of PON1 activity. In PON1 studies using plasma, selecting different types of PON1 activities may lead to yield inconsistent results. However, we demonstrated the prevention of deactivation by calcium addition immediately after sample collection, suggesting that plasma can be used for measuring PON1 activity. Plasma PON1 proteins were stable and minimally affected by EDTA inhibition, as the PON1 activity did not decline for one week after calcium supplementation. This finding is beneficial for HDL-PON1 study because PON1 transfers from HDL to the oxidized LDL which is derived by existence of copper [27]. Moreover, myeloperoxidase, which is known to be released mainly from neutrophils through the coagulation process, modifies HDL and inhibits the PON1 activity [38]. For this reason, the use of plasma also offers benefits as a non-coagulated sample. We suggest that plasma with calcium addition is a more suitable sample reflecting the *in vivo* PON1-related environment than serum.

Furthermore, our results showed that PON1 protein concentrations decreased in HDL isolated from plasma compared to those in HDL from serum. HDL collected from serum plus EDTA sample also had lower PON1 concentrations. Although data variability in the three experiments was observed because of the different recovery rates of PON1 concentrations, the HDLs isolated from serum plus EDTA and plasma, i.e. samples exposed to EDTA, showed low PON1 protein concentrations. These results suggest that the presence of EDTA, rather than the coagulation process, contributes to PON1 dissociation during ultracentrifugation. Thus, EDTA might promote the PON1 release during ultracentrifugation, weakening the PON1 anchoring to the HDL particle. PON1 is anchored to the HDL particle by an *N*-terminal α helix (H1) and subsequently the other short helices, H2 and H3, that are anchors for PON1 [4]. Calcium deficiency leads to irreversible structural changes in PON1, resulting in its inactivation. In addition, this changes in PON1 structure might alter the PON1 connectivity to the HDL particle. Free PON1 is unstable and activated by binding to HDL particle. Calcium chelation by EDTA may reduce PON1 activity not only by changing the structure of the active site but also of the binding site with HDL.

Further, we demonstrated that the PON1 binding was destabilized by ultracentrifugation even in HDL isolated from serum, which indicates that the ultra-centrifugal force damages the anchoring of PON1 to the HDL particle as shown in previous studies [27,39]. Thus, we revealed that the calcium-added plasma is an appropriate sample for blood PON1 assessment; however, further investigation on specimens for HDL-PON1 analysis was necessary because of weakened anchoring to HDL by calcium chelation.

Therefore, we next proposed BDS as a new specimen for targeting only HDL-PON1. BDS has been used for determining HDL functions such as cholesterol efflux capacity [22]; thus, we presumed that BDS could be used to evaluate only the HDL-PON1 activity and not the other lipoprotein-PON1 activities. Although slightly decreased, the PON1 activity and concentrations in BDS sample were more similar to those in serum sample than those in HDL sample. Moreover, in the WB profile of BDS, multiple PON1 bands at different molecular sizes were observed, which is consistent with the serum profile unlike that of the HDL sample (Fig. 4D). PON1 is known to bind to various proteins associated with HDL such as apoA-I and apolipoprotein A-II, and myeloperoxidase [20,40]. These multiple PON1 bands suggest different molecular forms or associations of PON1 in serum and BDS that are less pronounced or absent in isolated HDL. Those complexes were detached from the HDL particles, making HDL isolated by ultracentrifugation a specimen distinct from its *in vivo* environment.

Considering the slight difference in PON1 activity and concentrations between serum and BDS by individuals, small quantities of PON1 protein residing in chylomicron and VLDL were assumed to be precipitated at the bottom during the BDS preparation [21]. A previous study showed that VLDL decreased HDL-PON1 lactonase and arylesterase activities [41], which indicates that HDL-PON1 in serum sample might be affected more than that in BDS sample. The causes of PON1 hypofunction might be revealed by PON1 assessment of both of serum and BDS. We hypothesize that BDS, by selectively removing apoB-containing lipoproteins, could potentially reveal functional differences in PON1 (e.g., due to abnormal expression or structural modifications). These PON1 functions might be masked when assessing total serum PON1 activity, particularly in conditions with altered apoB-lipoprotein levels. BDS may be a suitable specimen for HDL-PON1 analysis, which reflects the *in vivo* environment of PON1 eliminating the influence of apoB-related lipoproteins.

Moreover, when compared to that in serum sample, the reduced PON1 activity in BDS showed variability with individuals, which might be caused by the lipoproteins other than HDL. Furthermore, because of the variability in the three PON1 activities, those activities might be affected differently by apolipoprotein B-containing lipoprotein. Overall, we expected that BDS might be a potential specimen to bypass the effects of apolipoprotein B-containing lipoproteins on PON1. To verify the presence of HDL-PON1 in BDS, we performed a separation of serum and BDS samples by Native-PAGE. WB profile results showed that the BDS sample preparation process did not affect the PON1 binding to the HDL particle despite the addition of PEG and centrifugation. Previous studies also reported that the PON1 activity and protein were present throughout the HDL fraction separating serum by high-performance liquid chromatography [20,42]. Our results were similar to those of previous studies and indicated that BDS might be a new specimen for searching HDL-PON1.

We suggest the importance of evaluating various types of PON1 activities because the three PON1 activities measured in this study showed different by individuals. However, among the various PON1 activities, selection of the suitable PON1 activity type for PON1 assessment has not been clarified. The blood native substrate has remained unknown, and we do not know whether all PON1 activities are related to the PON1 functions. For example, although homocysteine-thiolactone can also be a substrate in the body, there is another enzyme that is more active to it than PON1 [43]. PON1 could not hydrolyse homocysteine-thiolactone preferentially *in vivo*. Based on these facts and our results, further studies for sample preparation and activity type selection are required before deciding in favour of PON1 regarding its usefulness as risk factor for several diseases.

A limitation of our study is that we did not consider the effect of numerous single nucleotide polymorphisms (SNPs) in *PON1* gene on its activities. Because SNPs affect differently in each PON1 activity [44,45], investigation of the correlation of PON1 activities with their SNPs and suitable substrate for each type of SNPs are necessary while discussing individual variability and the applicability of BDS to basic research such as investigating the effects of modified HDL and lipoproteins on PON1. Moreover, we have not verified the effect of substances other than lipoproteins on the PON1 activity. Because BDS contains many major proteins such as albumin and globulin, they might affect PON1. Nonetheless, HDL-PON1 research using BDS could more accurately reflect the *in vivo* environment than serum, and BDS will be an important tool for accurate functional PON1 studies.

## 5 Conclusion

This study revealed the advantages of using plasma and BDS as specimens for studying PON1 activities. We demonstrated that EDTA plasma PON1 activity can be accurately measured with immediate calcium supplementation, providing a more suitable sample for comprehensive PON1 assessment by circumventing the effects of copper oxidation and released blood components. In addition, BDS sample can avoid effects of apolipoprotein B-containing lipoproteins on PON1 activity and be evaluated for PON1 remaining bound to the HDL particle. We expect to enable a more detailed PON1 evaluation using BDS sample along with serum or plasma. While BDS shows promise in reflecting the *in vivo* PON1 environment, further studies are warranted to fully establish its utility as a standard specimen for diverse PON1 studies.

## CRediT authorship contribution statement

**Akira Yoshimoto:** Writing – review & editing, Methodology.

## Funding sources

The author(s) disclosed receipt of the following financial support for the research, authorship, and/or publication of this article: This work was supported in part by a Grant-in-Aid for Scientific Research (C) from the Japan Society for the Promotion of Science [22K07466] to R.O.

## Declaration of competing interest

The authors declare the following financial interests/personal relationships which may be considered as potential competing interests: Ryunosuke Ohkawa reports financial support was provided by the 10.13039/501100001691Japan Society for the Promotion of Science. If there are other authors, they declare that they have no known competing financial interests or personal relationships that could have appeared to influence the work reported in this paper.

## Figures and Tables

**figs1:**
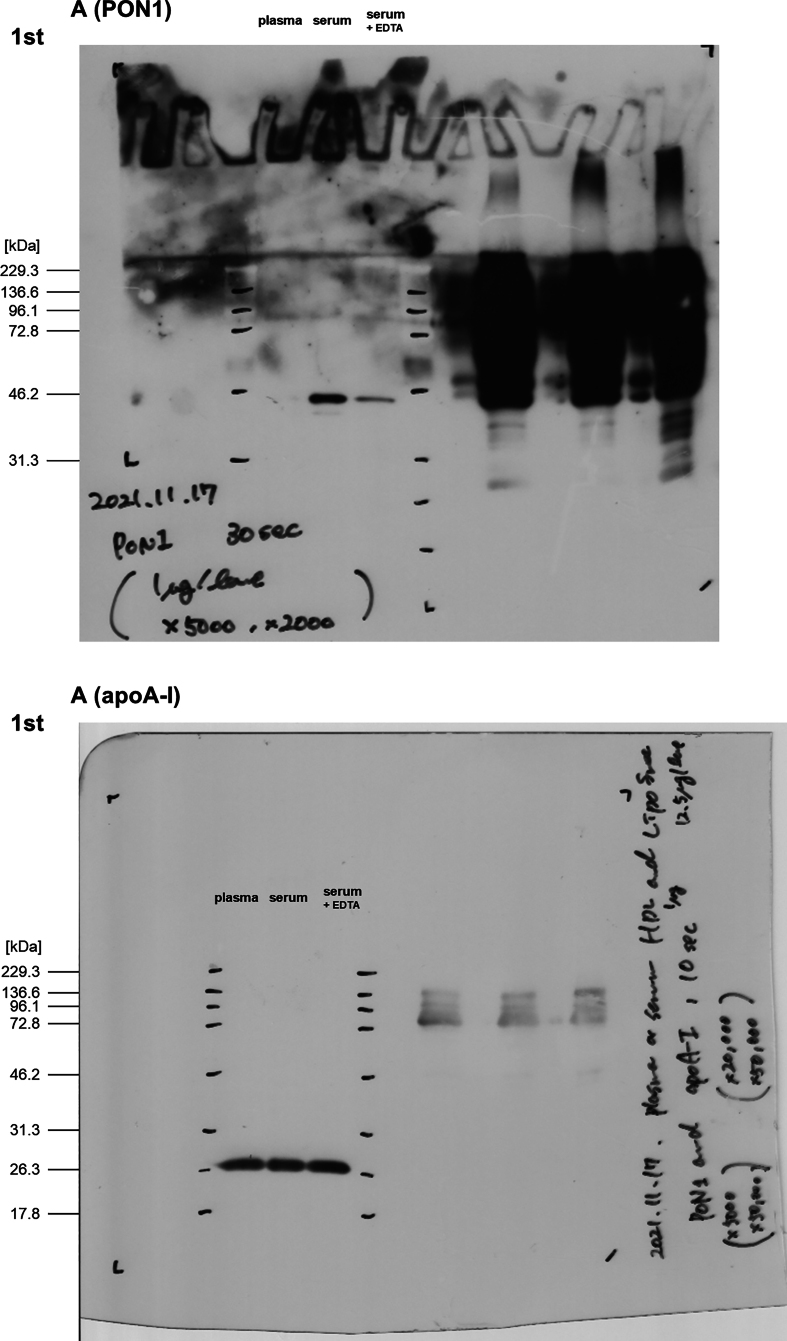


**figs2:**
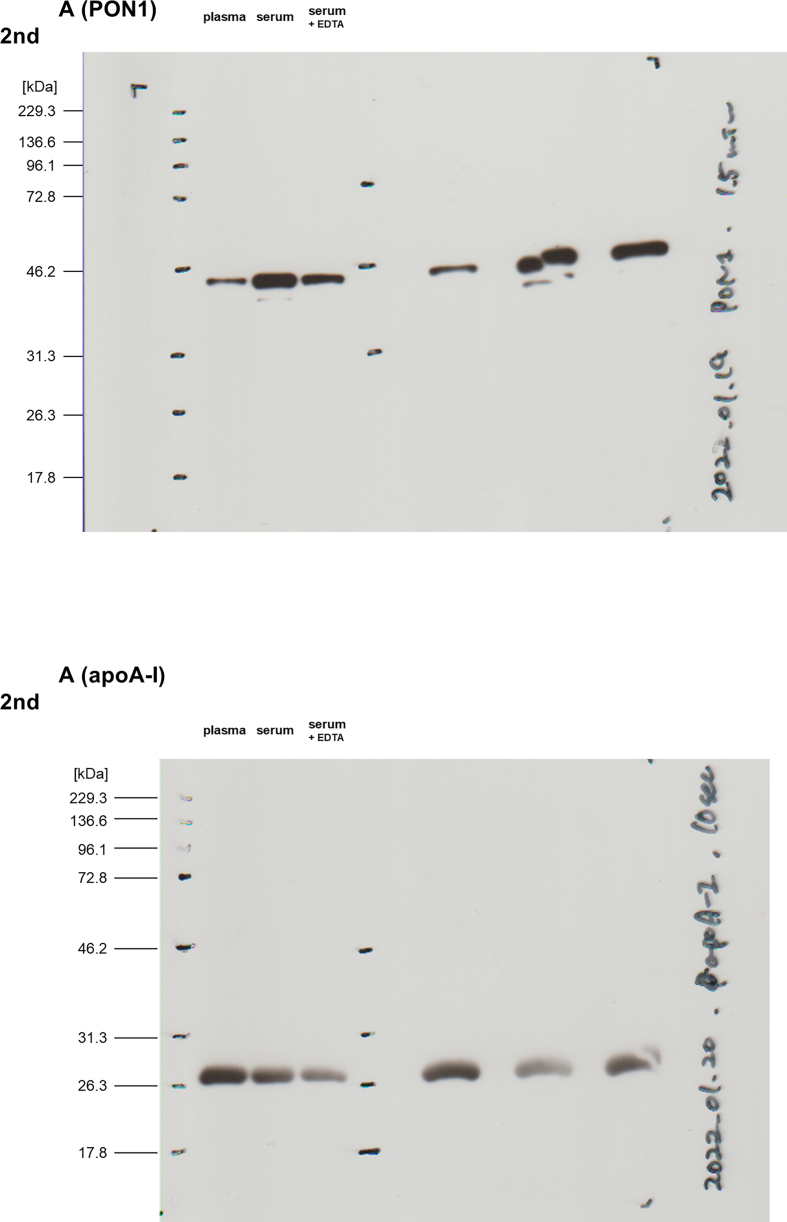


**figs3:**
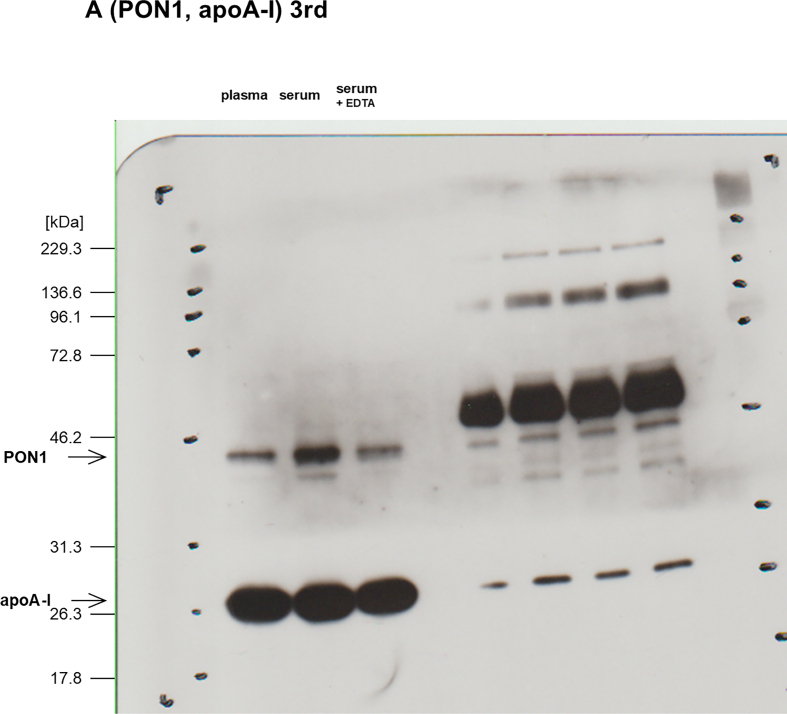


**figs4:**
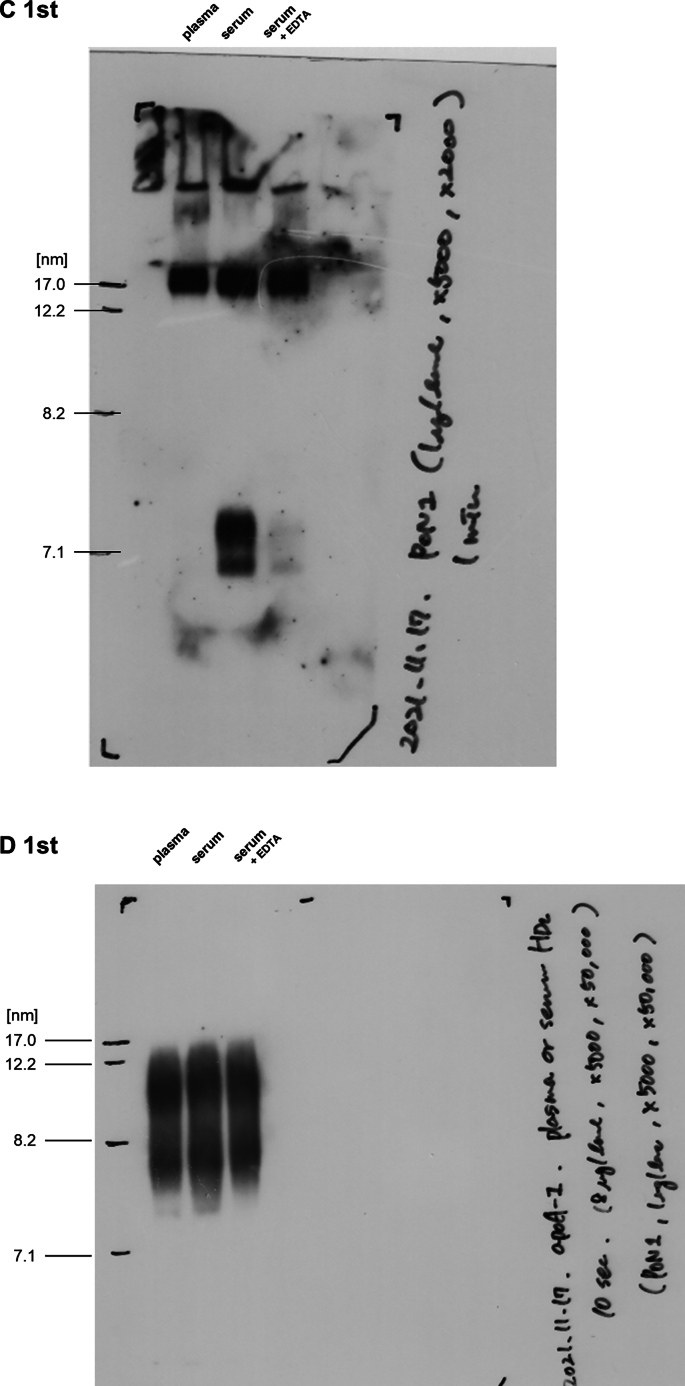


**figs5:**
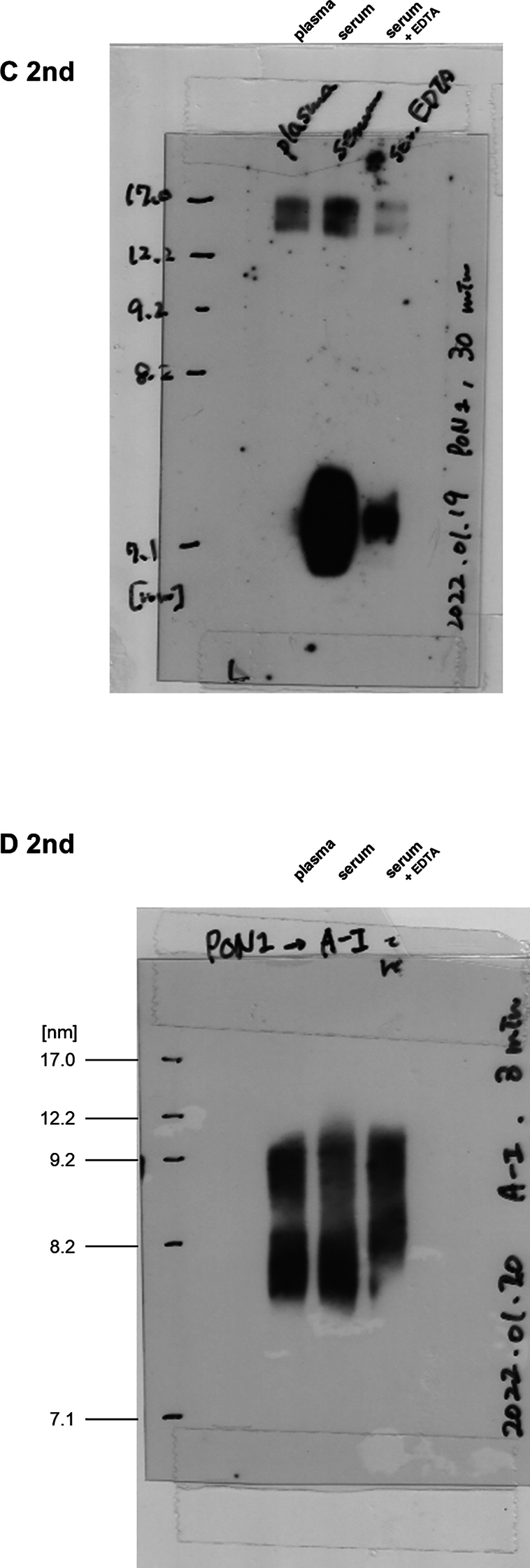


**figs6:**
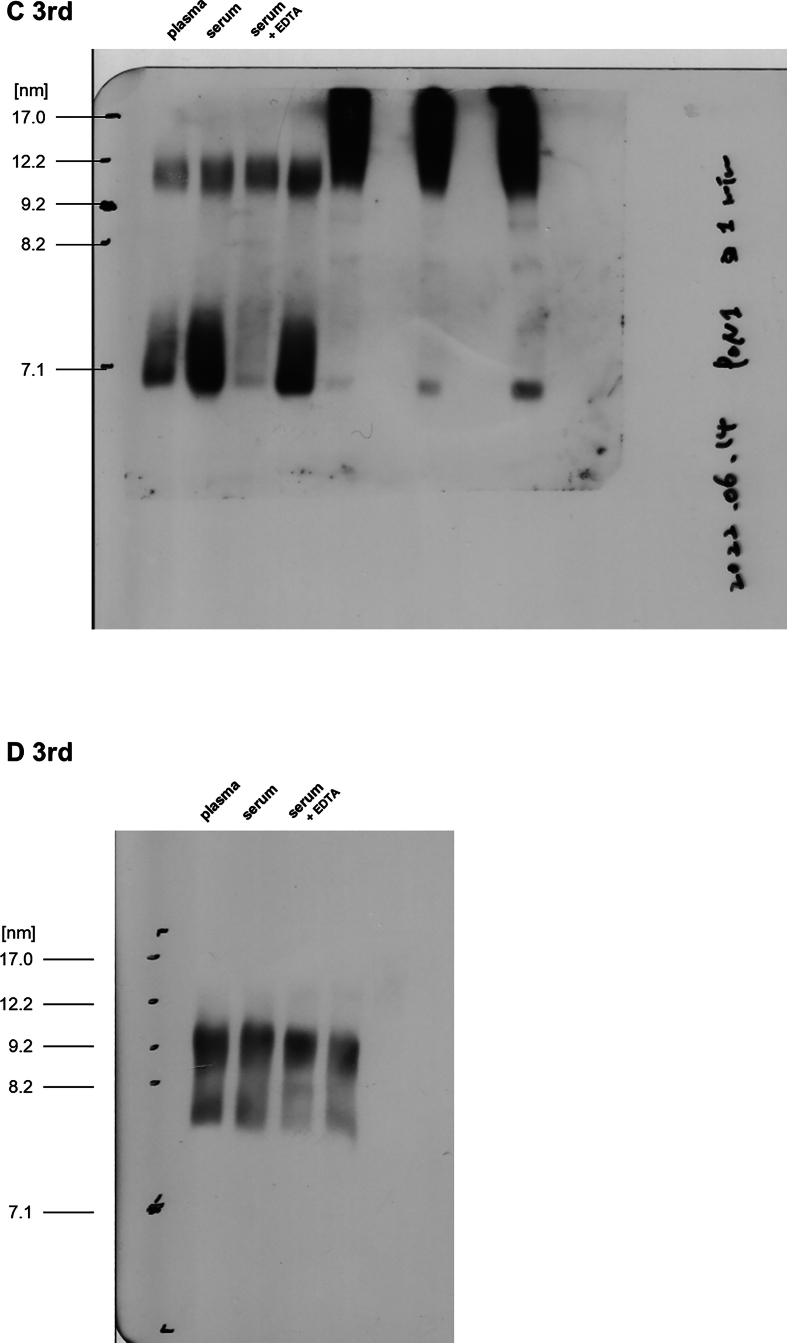


**figs7:**
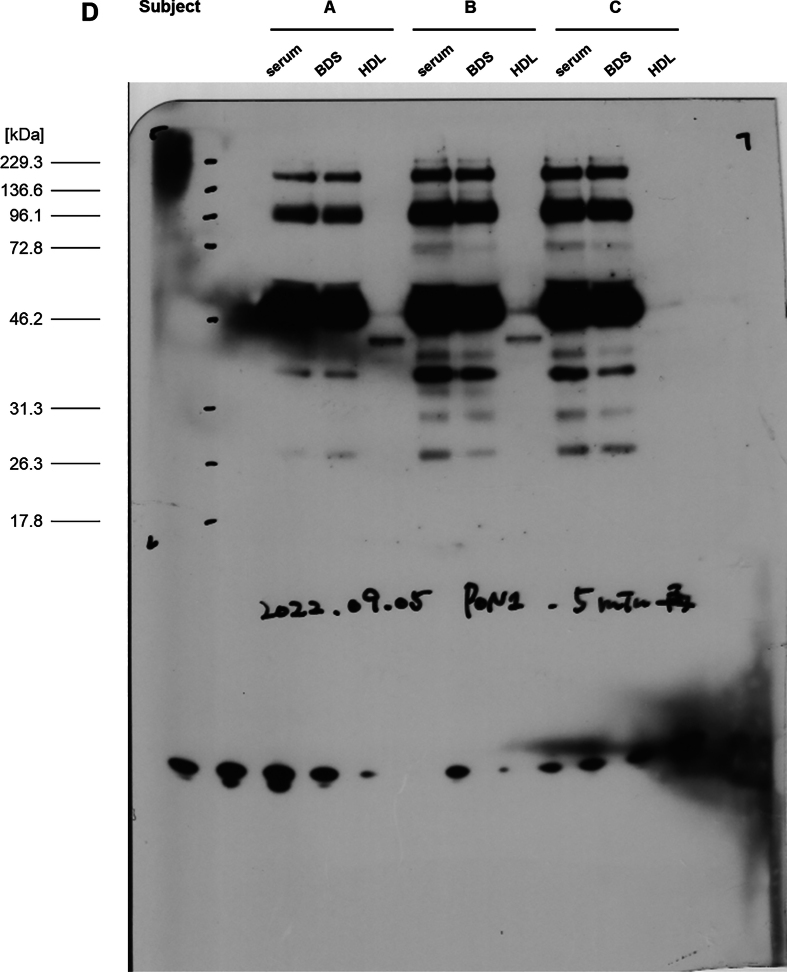


**figs8:**
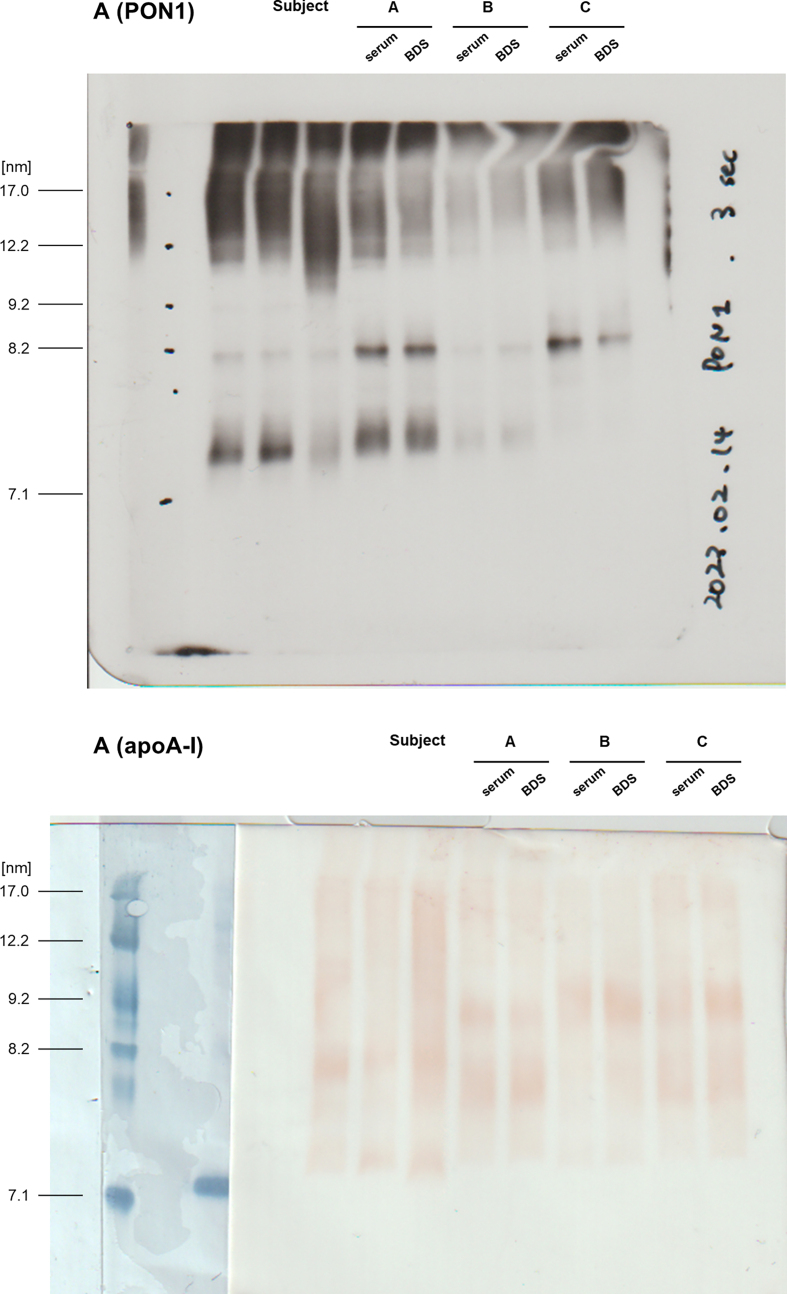


## Data Availability

Data will be made available on request.
